# Review of iodine behavior from nuclear fuel dissolution to environmental release

**DOI:** 10.1039/d4ra06494a

**Published:** 2024-11-08

**Authors:** Chelsie L. Beck, Juan Cervantes, Steven Chiswell, Allison T. Greaney, Katherine R. Johnson, Tatiana G. Levitskaia, Leigh R. Martin, Gavin McDaniel, Stephen Noble, Jason M. Rakos, Brian J. Riley, Andrew Ritzmann, Joel M. Tingey

**Affiliations:** a Pacific Northwest National Laboratory Richland WA 99354 USA chelsie.beck@pnnl.gov; b Oak Ridge National Laboratory Oak Ridge TN 37830 USA; c Savannah River National Laboratory Aiken SC 29831 USA; d ENSCO Vienna VA 22180 USA

## Abstract

During nuclear fuel reprocessing, radioiodine, can be released. The speciation of iodine drives its volatility, and partitioning processes are highly variable because they depend on facility operating conditions. Starting from iodine behavior in the fuel and progressing to its behavior in the environment, this review article describes the current understanding of iodine partitioning during aqueous fuel reprocessing. This review outlines knowledge gaps and describes the effects of state-of-the-art reprocessing techniques on iodine speciation and volatility. Whereas many review articles have described iodine behavior during specific reprocessing steps, this review provides a holistic overview of radioiodine, from the forms of iodine in different types of irradiated fuel to the forms of iodine released into the environment. The resultant behavior of radioiodine compared with stable iodine in the environment is also described.

## Abstract 2

During nuclear fuel reprocessing, radioiodine, can be released.

## Graphical abstract

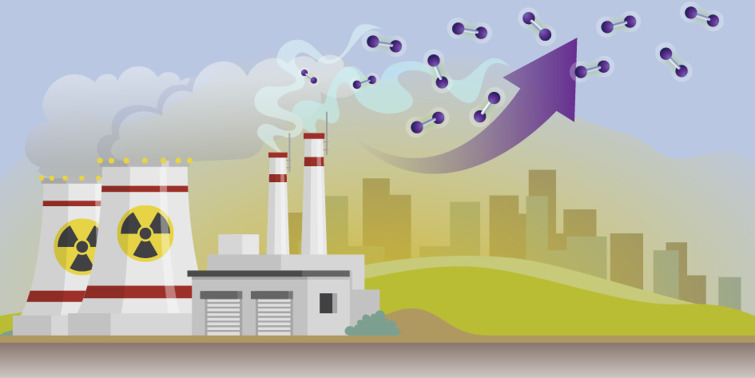

## 1 Introduction

**Fig. 1:**
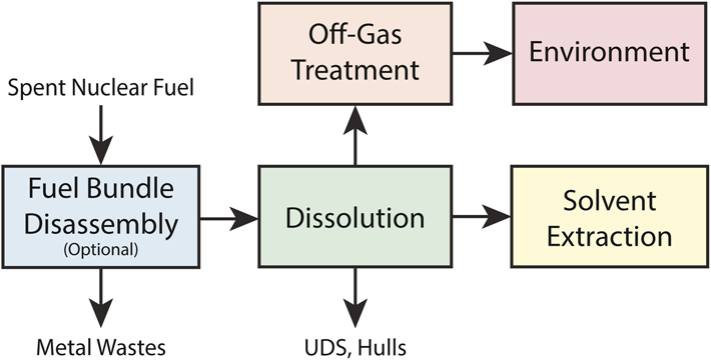
Schematic of process steps that are covered in this review. Fuel bundle disassembly is covered in Section 2, dissolution and off gas treatment in Section 3, solvent extraction in Section 4, and environment in Section 5.

Radioiodine is a highly regulated fission product that can be released during reprocessing of spent nuclear fuel (SNF). Iodine exhibits complex behavior driven by its many possible oxidation states and high reactivity. During fuel reprocessing, the volatility of the iodine determines the quantity of iodine released from the facility. This review article focuses on the current understanding of iodine partitioning during each reprocessing operation (as outlined in Fig. 1) and the conditions under which they are observed. The mass and activity of iodine in the fuel varies depending on irradiation conditions, which are not the focus of this review article; therefore, in this article, the irradiation of nuclear fuel is only discussed when it affects iodine speciation. Both oxide and metal fuels are discussed in this article. Oxides include ceramic UO_2_ fuels, which are commonly used in light-water reactors (LWRs) and pressurized heavy-water reactors (PHWR), as well as mixed uranium and plutonium oxide (MOX) fuels, which are used in fast reactors. Metal fuels have been used in both gas- and water-cooled graphite moderated reactors and fast breeder reactors. The fuel material and cladding impact processing conditions which in turn impact iodine speciation and volatility.

In reprocessing, one of the most problematic volatile radioisotopes is ^129^I. Other volatile radionuclides (*e.g.*^85^Kr, ^14^C, and ^3^H) are released during reprocessing but are not as hazardous as ^129^I. The long half-life of ^129^I (*t*_1/2_ = 1.57 × 10^7^ years), its complex reactivity and the health concerns resulting from exposure, create the need for high decontamination factors to mitigate the release of ^129^I into the environment.^1,2^ However, understanding the complex chemistry is difficult and continues to be an active area of research.^3–7^

This paper discusses, factors that lead to the partitioning, speciation, and interdependence of physical, chemical, and radiological mechanisms. For additional information, readers are referred to previous literature cited throughout these sections. The primary differences between past reviews and the current review are that the current review aims to cover a more holistic overview of radioiodine behavior during reprocessing from iodine forms in irradiated fuel to environmental release as well as differences in environmental behaviors between stable iodine and radioiodine. Section 2 discusses contemporary nuclear fuel treatment and the processes that affect iodine chemistry. Section 3 investigates reactions and products observed during dissolution, as well as the complexities between scales in laboratory studies and facility environments. Section 4 discusses the distribution in iodine products in chemical phases through extractants, waste forms, and treatment steps. The movement of iodine throughout the nuclear fuels system ultimately results in potential cycling to the environment. Section 5 presents general transformation mechanisms characterizing reactions, and interactions with the environment. Section 6 summarizes these topics as the basis for a comprehensive model for the iodine system.

## 2 Iodine chemistry during fuel disassembly

### 2.1 Fuel types

A large portion of research focuses on UO_2_ fuel because it is the most common fuel type used worldwide. A variety of reactors, including (LWR's), boiling water reactors, pressurized water reactors, pressurized heavy-water reactors (PHWR's), fast-neutron reactors, advanced gas-cooled reactors, and high-temperature gas-cooled reactors can use UO_2_ fuel. This section focuses on iodine behavior in UO_2_ fuels in LWRs and PHWRs. Fuel cladding is an important consideration because it impacts the disassembly process chosen. Both reactor types use zirconium alloy (Zircaloy) cladding. Alternative cladding materials, such as those used in accident tolerant fuels are beyond the scope of this review.^8,9^ Metal fuels are often clad in magnesium alloy (magnox), as was done in gas cooled reactors, or with aluminum or stainless steel as was done in water cooled reactors.

### 2.2 Head-end processing for UO_2_ fuels

**Fig. 2:**
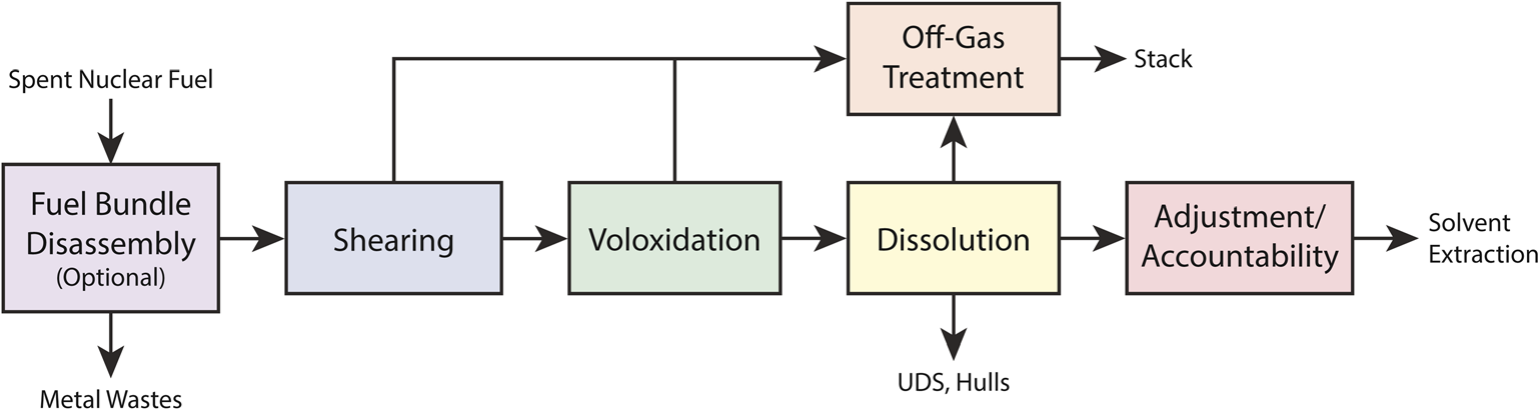
Block flow diagram for aqueous head-end processing.^10^ This figure was reprinted with permission. Copyright 2008 CRESP.

An advanced head-end process for uranium oxide fuel dissolution is shown in Fig. 2.^10^ Typical oxide fuel dissolution is similar but does not include voloxidation. The first head-end process is to remove the components of the bundle or assembly so individual fuel pins can be processed. Before fuel dissolution can occur, the cladding must be breached or removed from the fuel either mechanically or chemically. Mechanical decladding is typically performed for UO_2_ fuels and has the potential to release any volatile iodine that may have migrated to the gap between the fuel and the cladding. For Zircaloy or stainless-steel clad fuels, the fuel pins are sheared into small pieces to provide access to the uranium fuel for chemical dissolution. After shearing, voloxidation (O_2_-based) or advanced voloxidation (NO_2_-based) can be used to oxidize UO_2_ fuel to U_3_O_8_ powder and release volatile radionuclides.^11,12^ Both methods target the liberation of volatile radionuclides such as ^129^I, ^85^Kr, ^14^C, and ^3^H. These methods may be advantageous because they capture the vast majority of iodine in the head-end abatement systems and do not fractionate it throughout the reprocessing facility. Depending on how voloxidation is accomplished (including the temperature and gases used) a significant portion of iodine may be released from the fuel.

Most reprocessing facilities currently move straight from shearing to dissolution. Many publications report the fraction of iodine that is volatilized to the dissolver off-gas (DOG) but exclude the shearing off-gas, which is often combined with the DOG. The exclusion of the shearing off-gas creates difficulty in determining what fraction of volatilized iodine, if any, is released during shearing. Two studies at the Wiederaufarbeitungsanlage Karlsruhe (WAK) reprocessing plant in Karlsruhe, Germany, specifically measured the amount of iodine released during shearing and reported that 0.15–0.3% of the total iodine in the fuel was released during shearing.^13–15^

The amount of iodine released during head end processes is driven by the species of iodine in the irradiated fuel. Many factors affect iodine speciation and concentration in fuel. Compared with metal fuel, which has a small heat gradient, UO_2_ fuel has a large heat gradient between the center of the fuel and the outer edge. This large heat gradient leads to differences in the oxygen-to-metal (O/M) ratio at different locations in the fuel. The O/M ratio is important because it changes the thermodynamically favorable species. Iodine is expected to segregate in fuels but an understanding of speciation and the form remains understudied.^16^ In general, the Cs/I molar ratio is ∼10, which leads to the prediction that CsI would be the predominate species.^17^ Even though CsI has been found on the inner cladding of irradiated LWR, micro-drilling of LWR pins has indicated that iodine migrates slightly faster than Cs. This contradicts the idea that higher amounts of CsI form at the surface of the fuel.^18^ At lower temperatures, <1000 K, thermal diffusion of iodine is directly proportional to the fission rates.^19^ At higher temperatures, fission products migrate primarily *via* thermally induced diffusion in the UO_2_.^19^ The vast literature surrounding reactor accidents provides a reliable source of data for understanding iodine speciation in irradiated fuel.^20^ For fuel failure, specifically stress corrosion cracking of Zircaloy clad fuels, I_2_ does not directly cause the cracking. Rather the formation of ZrI_4_ (and potentially other lower forms of iodide) can result in cracking.^20^

Studies have shown that in PHWR reactors the cesium and iodine are held firmly in the fuel and sheath surface.^21^ This arrangement may be due to higher fuel temperatures, which, compared with the temperatures used for LWR fuels, lead to more fission gas segregation.^22^

Fuel burnup plays an interesting role in the speciation of iodine. At burnups of less than 5 MWd per kgU, CsI does not form at the grain boundary. Generally, burnup increases the fuel's O/M ratio, which, changes the favored species.^18^ The volatile species, including I_2(g)_, can accumulate in the annular region between the pellets and the cladding. A study at WAK showed that low- and high-burnup fuels have similar concentrations of undissolved iodine during fuel dissolution.^23^ Because more iodine is produced in high burnup fuels, this result indicates that in low-burn-up fuels, a higher portion of the iodine will be undissolved or released as gas than in higher-burn up fuels.^23^ Higher-burnup fuels cause iodine to be retained in a Ag–Pd halide phase, which is resistant to HNO_3_ dissolution.^24^ For high-burnup fuels, compounds of Ag, Pd and iodine have been measured, and the results indicate that these compounds were formed during irradiation and not during dissolution.^24^ Whether the same is true for low-burnup fuel still needs to be verified.

Stainless steel cladding is often used for MOX fuel. During reactor operation, iodine in MOX fuel is also expected to exist as CsI_(g)_, which can migrate to the fuel cladding gap. The CsI will not attack the stainless-steel cladding, but if I_2_ is formed from either radiolytic decomposition of CsI or from high O/M ratios, then I_2_ can attack the stainless steel and form metal iodides with the metals in the steel.^25–27^ A passivation layer further inhibits the ability of I_2_ to interact with the stainless steel, and an O/M larger than 2.08 is needed.^17^ In defective fuel, volatile fission products such as iodine, xenon, and krypton migrate to the defect site and eventually into the coolant.^28^

### 2.3 Head-end processing for metal fuels

Depending on the cladding material, metal fuels can be chemically or mechanically declad. Metal uranium fuel clad in magnesium metal alloy was used in gas-cooled reactors. The fuel was decanned at the cooling pond location before being transported to the dissolver. A report detailing the iodine controls during processing at Windscale, states that no significant release of iodine occurred during the decanning process.^29^ Therefore, if volatile iodine species do form in metal fuel, then the assumption is that they do not migrate significantly to the surface of the fuel.

Metal uranium fuel clad in aluminum has also been used. The aluminum cladding is chemically separated from the fuel by dissolving the cladding in NaOH with NaNO_3_ added to control hydrogen evolution.^30^ As with mechanical decladding, very little iodine was released during dissolution of the cladding; however, measurements performed at facilities indicated that some iodine was evolved during this step.^30^ Limited information is available on iodine characterization in metal fuel. Instead, some information on the species can be inferred based on volatility during dissolution. Speciation during dissolution is discussed in Section 3.

## 3 Aqueous dissolution

### 3.1 Overview

The chemistry of iodine during the dissolution of UO_2_ and metal fuel is covered in this section, which focuses on HNO_3_ dissolution. As discussed in Section 2.2, UO_2_ fuel elements are sheared to the desired length and are dropped into a basket in the dissolver, resulting in the release of a small fraction of the iodine. Concentrated (∼11 M) HNO_3_ is added to dissolver and heated to near boiling. In a facility-scale dissolver, the sheared pieces of fuel cladding are typically added to rotating baskets submerged in HNO_3_. Before chemical separation, the uranium oxide fuel is dissolved away from the cladding hulls and the dissolver is routed to a tank for nuclear material accountability and chemical adjustment. Metal fuels can be mechanically or chemically declad but are then dissolved in HNO_3_ similar to UO_2_ fuels.

### 3.2 Chemistry in the dissolver

The majority of iodine in UNF is assumed to be in the form of CsI,^31,32^ which is highly soluble in aqueous media owing to its low crystal lattice energy: CsI solubility in water is 85.5 g/100 g H_2_O at 25 °C. This value increases with increasing temperatures to >205 g/100 g H_2_O at 100 °C.^33^ Like other alkali iodides, CsI is soluble in HNO_3_ at a range of concentrations and temperatures,^11,34–37^ where the following reaction occurs.

1 2CsI_(aq)_ + 4HNO_3(aq)_ + 2H_2_O_(l)_ ↔ I_2(aq)_ + 2NO_2(aq)_ + 2CsNO_3(aq)_ + 4H_2_O_(l)_

Reaction (1) is a simplistic reaction and does not show that CsI must first disassociate to Cs^+^ and I^−^. The reaction also does not show the additional reactions that can occur, including the oxidation of I^−^ to IO_3_^−^ and the formation of compounds such as PdI_2_ and AgI which are discussed in Section 3.5. Thermodynamic data are available for the formation of these species; however, kinetics drive many of these reactions, and additional work is needed to fully understand this system. Although dissolution of I_2_ crystals in water is not favorable (3.4 g/100 g H_2_O at 25 °C),^38^ many reports support the solubility of I_2_ crystals in concentrated HNO_3_ solutions (3.12 mmol L^−1^ 69.95% HNO_3_ at 25 °C).^38–40^ The volatility of iodine from aqueous solutions is a known route for the release of iodine from the dissolver solution.^5^ The primary volatile species is assumed to be I_2_ but HOI and some organic iodides are also volatile. Several studies that monitored the dissolution of alkali metal iodides (*e.g.*, KI) in concentrated nitric acid solutions have confirmed that the reaction occurs rapidly,^11^ and is proportional to the rate of fuel dissolution when tested within a fuel matrix.^37^ The low concentrations of iodate ions (IO_3_^−^) are also expected, and can be further reduced to I_2_ by available NO_2_ species except at very low pH ([HNO_3_] > 16 M).^34^ However, studies from Cathers and Kibbey, which are documented in a report by Unger *et al.*,^41^ suggest that iodate ions are easily reduced to I_2_ as the concentration of HNO_3_ decreases to a concentration range (∼3 M) consistent with fuel dissolution. The primary reactive species of iodine (I^−^ and I_2_) form organic iodides (*e.g.*, CH_3_(CH_2_)_*x*_I where *x* = 0–12) or other volatile products, and their distributions in later processing steps depends on the total concentration of iodine.^37,42,43^

Experiments have shown that >90% of the molecular iodine is volatilized when UNF is dissolved in HNO_3_ with the addition of air sparging.^44,45^ Oxidation reactions of iodide in HNO_3_ with the resulting HNO_2_ content assist in the release of iodine from the dissolver solution. The oxidation reactions of iodide by HNO_3_ and HNO_2_ are shown in reactions (2) and (3). As shown in reaction (4), aqueous molecular iodine then transitions to gaseous iodine, which is swept out of the dissolver by the sparge air.^46^

2 I^−^ + 4H^+^ + NO_2_^−^ + 2NO_3_^−^ ⇌ 0.5I_2_ + 3NO_2_ + 2H_2_O

3 I^−^ + 2H^+^ + NO_3_^−^ ⇌ 0.5I_2(aq)_ + NO_2_ + H_2_O

4 I_2(aq)_ ⇌ I_2(g)_

For metal fuels, Burger reported that elemental iodine is the most stable iodine species in the dissolver solution (3–10 M HNO_3_), and that all iodine should eventually evolve as I_2_. The only exception occurs in solutions with high HNO_3_ concentrations (>10 M). In these solutions, iodate is the main iodine species present.^30^ Later studies showed that elemental iodine can be further oxidized to iodate in HNO_3_ solutions. The equilibrium reaction between elemental iodine and iodate in HNO_3_ solutions is shown in reaction (5).^47^ Sakurai initially believed that iodate was the main species remaining in solution after the volatilization of elemental iodine; however, his research group found that in solutions of 4 M HNO_3_, the main species of iodine remaining in solution is colloidal iodine.^47^ Gaps in the understanding of the composition of iodine remaining in solution still exist, and the actual iodine composition is clearly more complex than Burger or Sakurai described.

5 I_2(aq)_ + 8H^+^ + 10NO_3_− ⇌ 2IO_3_− + 10NO_2(g)_ + 4H_2_O

### 3.3 Alternative acids

Some studies investigated replacing HNO_3_ with HCl. These studies cite several advantages, including better separation and dissolution of fission products, recyclability, and better control of oxidation states. Mailen and Bell^48^ point out that, although the iodine compounds (in the form of I_2_, CsI, and RbI) are soluble, iodine volatilization from HCl may be additionally troublesome because of the formation of a highly reactive interhalogen species, the I_2_Cl^−^ ion.

### 3.4 Radiolysis in dissolution

The chemistry of iodine during UNF dissolution involves numerous chemical processes. Dissolution involves high concentrations of HNO_3_ which lowers the pH and introduces a powerful oxidizing agent (the nitrate anion). The ionizing radiation that results from the radioactive decay of the radioisotopes in the dissolver involves a complicated web of chemical reactions.^49,50^ The radioactive decay emits energy into the solution in the form of β particles, α particles, and γ-rays. The ionizing radiation from the radioactive decay causes radiolysis, the breaking of the chemical bonds of the molecules in solution and the production of several reactive species. The β particles and γ-rays emitted by decay of the fission products are expected to provide most of the dose rate within the solution, but the energy from α decay is deposited in a short distance and contributes significantly to radiolysis in the dissolver solution.

Species in solution may undergo direct radiolysis by interacting with a photon or β particle or may undergo secondary reactions with direct radiolysis products. The radiation energy deposited in the solution is generally distributed according to the electron fraction of each species.^51^ This quantity is derived from the mole fraction (or concentration) of each species along with the number of electrons in the species (*e.g.*, 10 electrons for H_2_O, 32 electrons for HNO_3_). Because of the low concentration of iodine present in the dissolver solution, its electron fraction is essentially zero and direct radiolysis may be neglected; however, the direct effect of radiolysis on water and nitric acid is relevant to the chemistry of iodine in the dissolver solution.

The conversion of dose rate to the number of radicals formed is described using a quantity called the *G* value. For pure H_2_O, the radiolysis products and corresponding *G* values are provided in Table 1. The immediate products include solvated electrons (e_aq_^−^), hydrogen (H˙) and hydroxyl radicals (OH˙), protons (H^+^), hydroxide ions (OH^−^), molecular hydrogen (H_2_), and hydrogen peroxide (H_2_O_2_). These products further react with water and each other to form numerous other species (*e.g.*, O^−^, O_2_, O_2_^−^, O_3_, O_3_^−^, HO_2_^−^, 
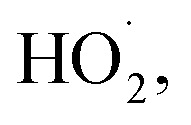
 and HO_3_).^52^ Exposures on the order of an hour or longer lead to a quasi-steady state distribution of radiolysis products. In this state, the most stable species (H_2_, O_2_, and H_2_O_2_) accumulate to appreciable levels.

**Table 1:** *G* values (in molecules per 100 eV) for γ-irradiation of water from Pastina and LaVerne^52^ as well as from HNO_3_ from Jiang *et al.*^51^ Part of this table was recreated with permission from Pastina and LaVerne.^52^ Copyright 2001 American Chemical Society

Species	Source	*G* (molecules per 100 eV)	*G* (μmol J^−1^)
e_aq_^−^	From H_2_O	2.60	0.27
H˙		0.66	0.068
H_2_		0.45	0.047
OH˙		2.70	0.28
H_2_O_2_		0.02	0.002
H^+^		3.10	0.32
OH^−^		0.50	0.052
NO_3_˙	From HNO_3_	4.8	0.50
NO_2_^−^		1.5 from NO_3_^−^	0.16 from NO_3_^−^
		2.0 from HNO_3_	0.21 from HNO_3_

The HNO_3_ undergoes direct radiolysis to form two reactive derivatives: 
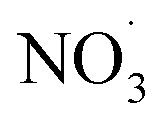
 and NO_2_^−^. In acidic solution, the nitrite will rapidly protonate to form nitrous acid, which undergoes further decomposition to NO_2_ and NO.^53^ The nitrate radical, 
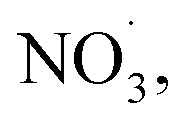
 subsequently breaks down to form 
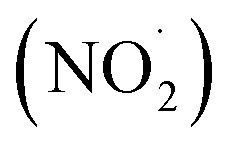
 and (O˙). The highly reactive O˙ then extracts an oxygen atom from NO_3_^−^ to produce O_2_ and NO_2_^−^.

Direct radiolysis products along with the radicals produced by subsequent reactions have extensive chemical interactions with iodine in the dissolver solution.^49,50^ First, I^−^ is oxidized into I_2_ by the presence of the OH˙ and 
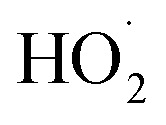
 radicals. The I_3_^−^ specie appears as an intermediate because dissolved I_2_ readily associates with I^−^.^54^ Once the iodine has been oxidized into I_2_, two phenomena may occur, the I_2_ may volatilize or it may undergo further oxidation to form HOI and HIO_3_. The interplay between vapor evolution and oxidation depends on the conditions within the dissolver, the time over which the dissolution occurs, and the time over which the diffusion through the solution into the headspace occurs.

### 3.5 Insoluble products from dissolution

Small fractions of UNF may not completely dissolve under the acidic conditions described previously. UNF can form solid aggregates, colloids, microspheres, or sludge within the dissolver vessel. The total amount and composition of insoluble residue that remains from the dissolution process varies based on fuel type, burnup, and length of storage.^11,55^ Elements that remain in this undissolved material include ruthenium, molybdenum, technetium, rhodium, palladium, and iodine.^56^ In the 1990s, Sakurai pioneered isolated studies of iodine interactions with fission products to provide a better understanding of this reaction mechanism.^47,57^ By reacting solubilized iodides with Ag and Pd, Sakurai created colloidal species in the forms of AgI and PdI_2_.^47,57^ Similar studies using copper, aluminum, and zinc as reactive surfaces also lead to the formation of insoluble metal iodides.^58^

In an effort to further study the speciation in the dissolved fuel, Sakurai *et al.*^45^ dissolved ^131^I labelled KI and UO_2_(NO_3_)_2_ in 3.4 M or 6.1 M HNO_3_, with and without the presence of Ag and Pd among other fission products. In the 3.4 M HNO_3_–UO_2_(NO_3_)_2_ system, they found that the major iodine species remaining in solution was iodate, but only at NO_2_ pressures of less than 3 × 10^−2^ atm and the pressure in dissolved fuels is expected to be >7 × 10^−2^ atm.^59^ However, the simulated UNF system containing fission products produced unknown iodine colloids. Furthermore, the quantity of these colloids increased with increased concentration of Ag and Pd. Therefore, it seemed likely that in dissolved fuel the major iodine species would be colloidal and not iodate,^45^ these results were further confirmed in subsequent studies.^47,59^

Sakurai *et al.*^47,60^ further tested the colloids hypothesis by dissolving used fuel pellets and actual fuel specimens. From these studies, Sakurai concluded that until the amount of iodide decreases to less than 10^−10^ M, the amount of Ag, Pd, and I^−^ present in dissolved fuel is sufficiently high that the formation of colloids is possible. The researchers confirmed that colloidal species are the most prominent form of iodine in dissolved fuel because iodide is initially on the order of 10^−5^ M in dissolved fuel.^46,47^ Non-volatile organic iodides are theorized to form as a results of organic impurities in HNO_3_.^61^ The primary organic impurity is CH_3_I, which is hypothesized to be able to undergo a similar decomposition reaction as colloidal iodine.^7,61^

**Fig. 3:**
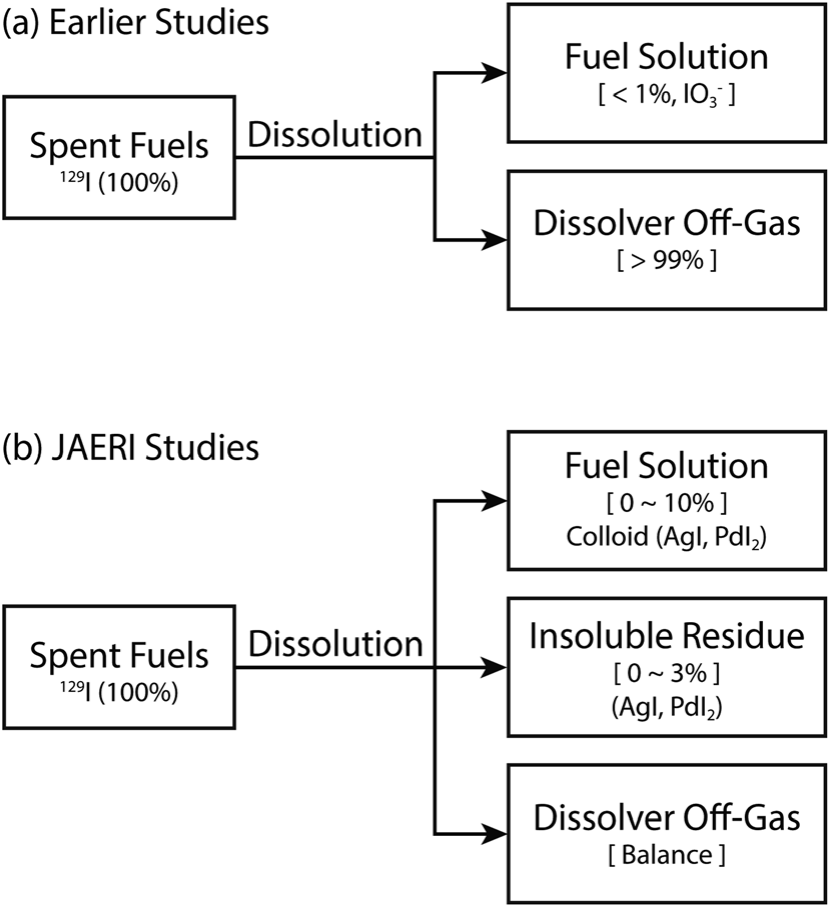
Distribution of iodine (^129^I) from dissolution of spent fuels in laboratory scale experiments. This figure was recreated from Sakurai *et al.*^46^ and was reprinted with permission.

In used nuclear fuel solutions, there is a retention of iodine species, which are suggested to be a mixture of I_2(aq)_, IO_3_^−^, and colloidal iodine. Colloidal iodine is produced by reaction (6)–(10),^46^ however, multiple additional metals in solution could also form iodine species. Because these known reactions, researchers modified early studies to account for remaining iodine species, as shown in Fig. 3.^46^ The updated studies rely upon the iodine behavior that was present during a lab-scale demonstration dissolution at the Japan Atomic Energy Research Institute. This demonstration suggested that ≤10% of the total iodine from dissolution could be in the fuel solution, ≤3% being in insoluble residues, and the remaining being off-gasses as molecular iodine (*i.e.*, I_2(g)_).

6 AgI_(s)_ + 2H^+^ + NO_3_− ↔ 0.5I_2(aq)_ + Ag^+^ + NO_2(g)_ + H_2_O

7 PdI_2(s)_ + 4H^+^ + 2NO_3_^−^ ↔ I_2_ + Pd^2+^ + 2NO_2(g)_ + 2H_2_O

8 5AgI_(s)_ + HIO_3_ + 5HNO_3_ ↔ 3I_2_ + 5AgNO_3_ + 3H_2_O

9 5PdI_2(s)_ + 2HIO_3_ + 10HNO_3_ ↔ 6I_2(aq)_ + 5Pd(NO_3_)_2_ + 6H_2_O

10 3I^−^ + Ag^+^ + Pd^2+^ ↔ AgI_(s)_ + PdI_2(s)_

Contaminated aqueous streams may also inhibit the complete dissolution of iodine in fuel. Although a study by Castleman and coworkers^55^ ruled out water impurities, Lieser and coworkers^36^ allude to potential causation in HNO_3_ purity. It is also difficult to dissolve colloidal iodine species even after NO_2_ sparging with concentrations remaining as high as 20 μg I/100 mL.^59,62^ A clarifying filtration step to remove these particulates may be necessary.

### 3.6 Partitioning

The complexity of iodine chemistry allows it to partition into gas, solid, and liquid phases during the dissolution of UNF in HNO_3_. Iodine partitions into the gas phase as volatile inorganic and organic species; iodine partitions into the solid phase as undissolved colloidal species, which are typically associated with Ag or Pd.^15^ Therefore, if radioiodine is not completely removed from the dissolver, it will spread into other off-gas and liquid-based waste streams throughout the facility. Dissolved residual iodate moves into the solvent extraction vessel, where radioiodine may partition into the off-gas or build in concentration in the organic solvents. This radioiodine may interfere with the intended extraction processes as the solvent is recycled. Undissolved solids move into solid waste processing where iodine may volatilize during evaporation processes and may need to be abated. Because the environmental and health concerns surrounding the release of radioiodine into the atmosphere, significant research has been devoted to ensuring that nearly all iodine is released during the dissolution step so that all iodine is abated from the dissolver off-gas.

#### 3.6.1 Experimental iodine partitioning

Over the past three decades, experimental studies and observations from operating reprocessing plants have attempted to quantify the percentage and speciation of iodine that is volatilized into the dissolver off-gas. The work of Sakurai *et al.*^45,62^ suggest that during dissolution in 100 °C HNO_3_ (∼4 M), between 90 and 99% of the iodine inventory is volatilized into the dissolver off-gas as I_2_. In general, the dissolution in HNO_3_ and the evolution of gaseous iodine can be summarized by reaction (11).

11 2I^−^ + 4H^+^ + 2NO_3_− ↔ I_2_ + 2NO_2_ + 2H_2_O

Because only 90% of the UNF iodine inventory may be volatilized, up to 10% of the iodine may remain in the dissolver as dissolved iodate (IO_3_^−^) or colloidal iodide (AgI and PdI_2_).^62^ The reaction of iodine with the fission products Ag and Pd to form colloidal AgI and PdI_2_ is routinely observed in experiments, as shown in reaction (10).^45,60,62^ The quantity of colloidal species is likely determined by the amount of HNO_2_ in the solution. This HNO_2_ forms during the dissolution of UO_2_.^57^ As the dissolution rate increases, the concentration of HNO_2_ increases, thereby decreasing the mass of residual colloidal iodine species.^62^ Thus, a higher dissolution rate will likely yield more volatile I_2_.

To increase the amount of iodine volatilized from the solution, experimental studies have shown the need to sparge the dissolver solution with NO_*x*_ gas and add excess iodate. Sakurai *et al.*^45^ found that after the dissolution of fuel is complete, the addition of NO_2_ gas to the dissolution solution converts any remaining aqueous IO_3_^−^ to gaseous I_2_, as shown in reaction (12). Similarly, the addition of excess IO_3_^−^ as a strip gas may convert colloidal AgI and PdI_2_ to gaseous I_2_, as shown in eqn (8) and (9), respectively. This process allows for a near quantitative release of the UNF iodine inventory.

12 2H^+^ + 2IO_3_− + 10NO_2_ + 4H_2_O ↔ 10HNO_3_ + I_2_

These experiments were repeated by Boukis and Henrich^61^ who found that the dissolution step may take >15 hours to remove >99% of the iodine inventory, and that both NO_2_ sparging and KIO_3_ stripping are necessary to achieve this removal efficiency. In that study, NO_2_ was generated by sparging the solution with NO (1.2 L h^−1^) and O_2_ (0.2 L h^−1^) diluted in N_2_. Excess NO_*x*_ and HNO_2_ were removed from the dissolver with a subsequent air sparge.

Mineo *et al.*^63^ performed bench-scale dissolution tests of UNF to determine the percentage of radionuclides that were volatilized. The experiments found that, during dissolution at 90 °C in 4–5.5 M HNO_3_, 62–72% of the iodine inventory was released. After dissolution, the dissolver solution was stripped of iodine using a 2 hour KIO_3_ dissolution and 2 hour NO_2_ purge. The iodine stripping step likely released the residual dissolved iodine because no ^129^I was detected in the dissolver after this step, although the amount of iodine sequestered in solids could not be accurately quantified.

#### 3.6.2 Observed iodine partitioning

Observations of iodine partitioning into the dissolver off-gas during UNF dissolution in reprocessing facilities broadly confirm experimental findings. In WAK Karlsruhe in Germany, approximately 94% of iodine was released to the dissolver off-gas during reprocessing activities in the 1970s and 1980s, and the remaining ∼5% moved to the solvent extraction vessel.^13^ This dissolver release efficiency was achieved after >8 hours of dissolution at 100 °C and NO_2_ sparging. However, Henrich *et al.*^64^ and Herrmann *et al.*^65^ suggested that by adding a NO_*x*_ sparge and iodate carrier gas, WAK Karlsruhe could expel >99% of the iodine from the dissolver off-gas. Thus, a range of possible iodine release fractions have been observed in operating plants. These release fractions depend on dissolver conditions and analytical uncertainty.

### 3.7 Iodine speciation in the DOG

The primary volatile iodine species in the dissolver off-gas is elementary I_2_.^45^ However, organic iodoalkanes from methyl iodide to dodecyl iodide (*i.e.*, CH_3_(CH_2_)_*x*_I, *x* = 0–11) have been observed or theoretically calculated to form in the dissolver off-gas.^66^ These organic iodides may form *via* reactions with solvents in recycled HNO_3_, or potentially even reaction with impurities in clean HNO_3_.^15,67^ Additionally, inorganic species such as HOI and HI have been theorized to form, but these iodine species are difficult to measure directly in the off-gas.^67^

Volatile iodine in the off gas has the potential to adsorb to the ducting surfaces. Most surfaces are expected to be either 304 or 316 austenitic stainless steel. Under a variety of conditions, I_2_ has been shown to form metal iodides with these materials.^27,68,69^ Due to the high humidity in these systems, highly deliquescent metal iodides (*e.g.*, FeI_*x*_) are expected to readily dissolve. The HNO_2_ and HNO_3_ in the gas stream can then oxidize iodides back to I_2_ as shown in reactions (2) and (3), thereby enabling reversible reactions. However, not all metal iodides formed from the interaction between I_2_ and stainless steel are deliquescent and previous research has shown that the adsorption is not completely reversible.^25–27^ Ducting with lower humidity and NO_*x*_ could result in the adsorbed metal iodides being favored, which would act as a sink for volatile iodine in the system.

Volatile iodine that is not trapped *via* adsorption with metal surfaces must be captured and retained by the facility's abatement system so that environmental regulations for safe release can be followed. A variety of abatement systems have been used ranging from liquid scrubbing (*e.g.*, aqueous caustic scrubbing, molten hydroxide scrubbing, Iodox, Mercurex) to solid sorbent capture beds (*e.g.*, silver mordenite, Clariant AC-6120).^6,66,70–78^ Wet scrubbing methods reduce the NO_*x*_ and some forms of iodine; dry solid sorbent capture methods can be used separately or in tandem with wet methods.

## 4 Solvent extraction

**Fig. 4:**
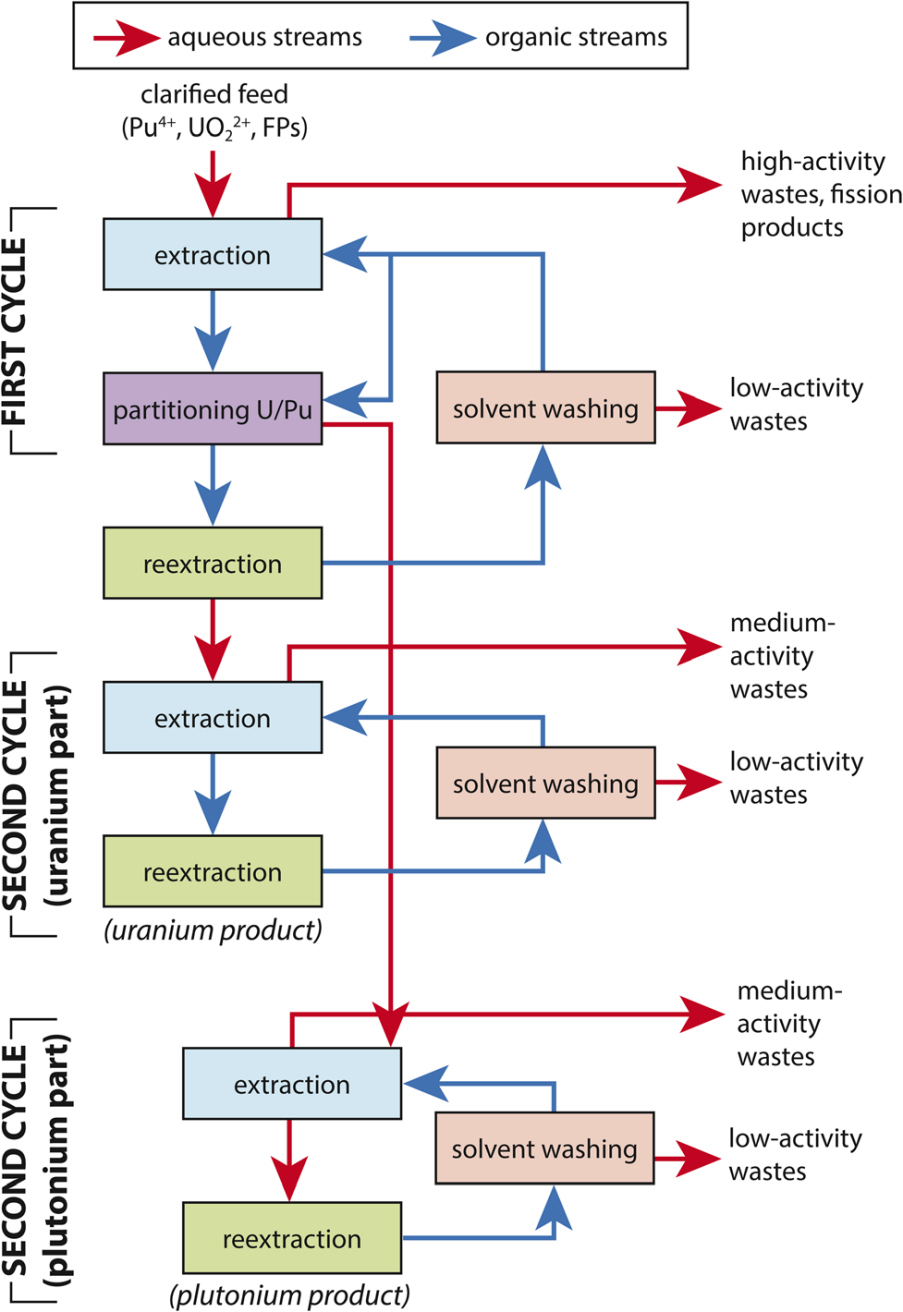
Traditional PUREX process.

**Fig. 5:**
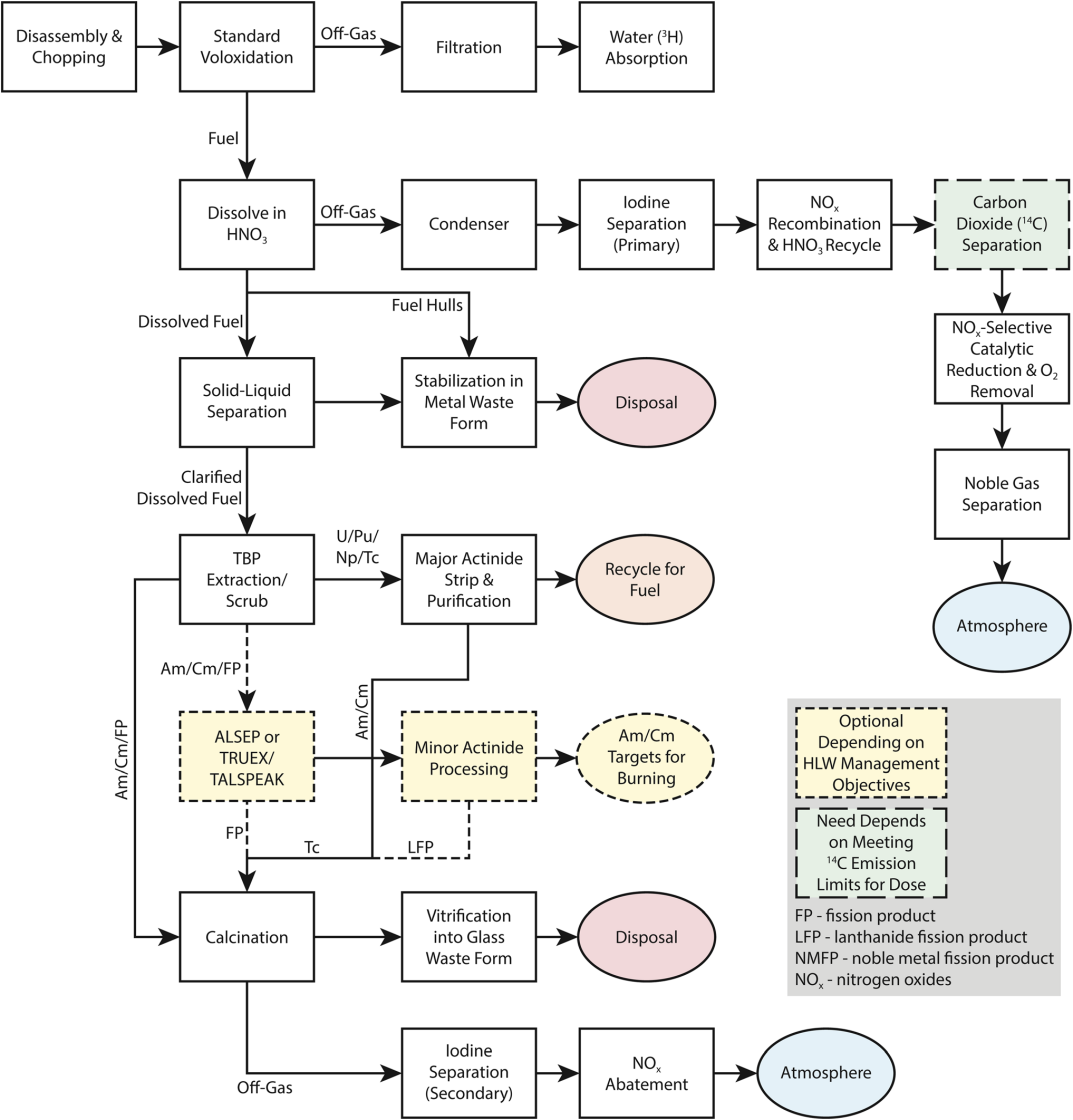
Basis of comparison flowsheet for UNF actinide recovery. This figure was recreated with permission from Arm.^79^

Throughout history, several extraction techniques have been utilized for the separation of lanthanides and actinides from systems. These techniques include the Plutonium Uranium Extraction Process (PUREX), Bismuth Uranium Extraction (BUTEX), Reduction Oxidation (REDOX), and Mercury Extraction. The most popular of these systems is PUREX, which is shown in Fig. 4. Possible modifications to the process are shown in Fig. 5.^79^ The remaining I species in solution from the fuel dissolution can reach these extraction phase processes and lead to several issues.^80^ For PUREX, the remaining I species are theorized to be a small amount of iodate, colloidal species, and organoiodides. The small fraction of iodate is formed through reaction (5).^15^ The distribution of I species leads to contamination in both the aqueous and organic phases. Roughly 40–50% of the iodine will transfer to the organic phase. This leads to contamination in both feedstocks. Additionally, owing to the recycling of solutions, an accumulation of I species can lead to buildup in wash solutions leading to higher activities, breakdown or interaction of the organic phase, and uncontrolled discharge to low activity waste lines.^7,15^

Because I^−^ oxidizes to I_2_ in the acidic fuel dissolution, the extraction of I_2_ with tributyl phosphate (TBP) has also been explored.^81–83^ Tsubomura and Kliegman^81^ reported the formation of the TBP·I_2_ in *n*-heptane with an absorption band at 460 nm. The band was attributed to the I_2_ bound to TBP, because free I_2_ absorbs at 525 nm.^81^ Tsubomura and Kliegman^81^ also stated that the I_2_ was likely bound to the oxygen in the P=O group in TBP rather than the R–O group. The oxygen in the P=O group is more negative than R–O, thereby allowing P=O to act as a better electron donor than R–O. The presence of a charge transfer band from I_2_ to the oxygen donor in TBP was also speculated to be below 250 nm.

Zagorets *et al.*^82^ also reported the formation of a TBP·I_2_ complex. They confirmed both the appearance of the bound I_2_ to TBP at 460 nm and the charge transfer band below 250 nm. They also suggested the decomposition of TBP·I_2_ through radiolysis and subsequent formation of I_3_^−^ through reactions (13)–(15).^84^ These low activity waste lines can lead to environmental contamination, as observed during fuel reprocessing at the Sellafield plan in the United Kingdom and the La Hague plant in France. At these sites, an estimated 1400 kg (Sellafield) and 3800 kg (La Hague), of radioactive iodine was released into the environment.^7,85^ Additionally, iodine insoluble residues build up in sediment and has an average of 2.34 × 10^9^ atoms per L of sediment.^86^

13 TBP + I_2_ ↔ TBPI_2_

14 TBPI_2_ ↔ TBPI^+^ + I^−^

15 TBPI_2_ + I^−^ ↔ I_3_^−^ + TBP

Historical observations of the exact fraction of iodine that remains in solution from fuel dissolution and eventually reaches the solvent extraction step have varied; however, all papers have reported some percentage of iodine being transported (even with extreme caution). For example, as reported by Henrich *et al.*,^36,87^ extensive treatment to remove the iodine from the dissolver solution by lowering the boiling temperature, and the addition of potassium iodate, still led to less than 1% of the iodine remaining with the recycled HNO_3_.^87^ In 1988, these results were further verified by Herrmann *et al.*^65^

## 5 Iodine transformation in the environment

### 5.1 General reviews

**Fig. 6:**
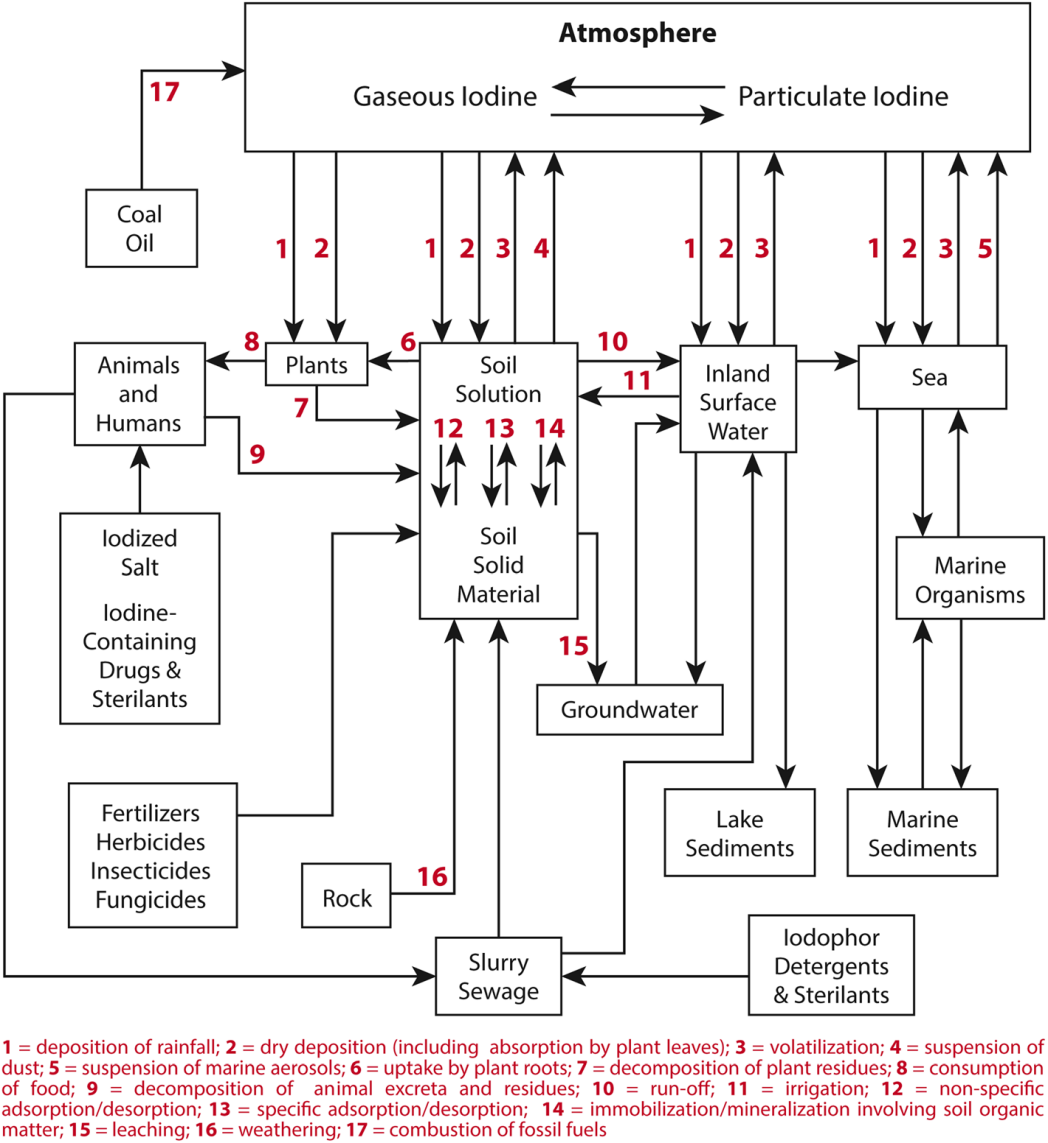
Outline of the movement of iodine in the environment. Figure was recreated from Whitehead and reprinted with permission.^88^ Copyright 1984 Elsevier.

Many references provide introductory material regarding the characteristics of iodine and its environmental cycling. Perhaps the most consistently cross-referenced is a paper by Whitehead,^88^ that provides a synopsis of iodine sources, concentrations, and general transformations throughout the atmosphere, lithosphere, hydrosphere, and biosphere. Fig. 6 provides a schematic of these processes and reservoirs.

**Fig. 7:**
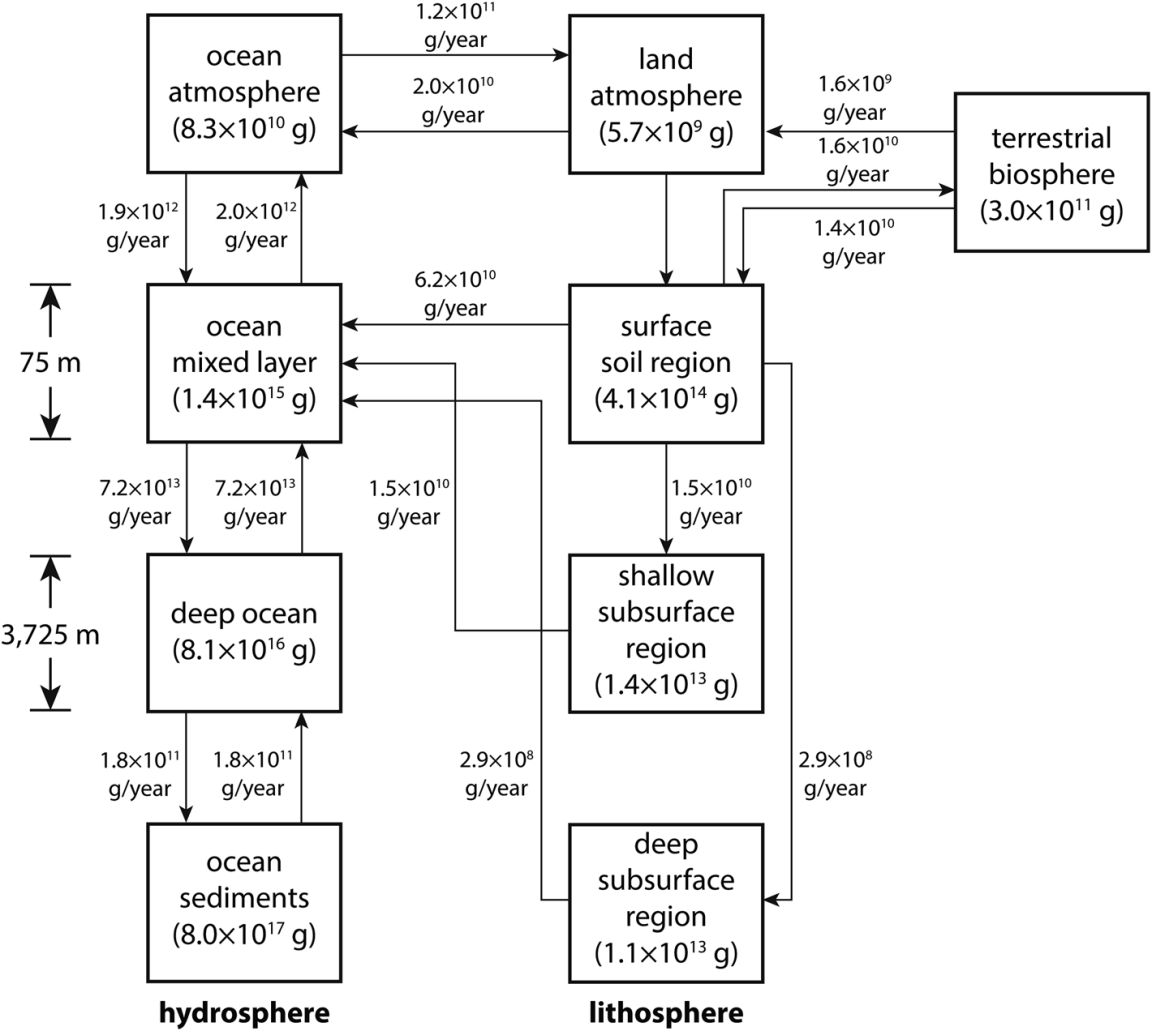
Diagram of the global iodine cycle at steady state that shows environmental compartment inventories in grams (g), transport pathways, and fluxes in grams per year (g per year). This figure was taken from ATSDR (2004).^89^

The 2004 Toxicological Profile for Iodine published by the Agency for Toxic Substances and Disease Registry (ATSDR) is a large volume (580 pages) that describes the effects to human health that result from exposure to various isotopic forms of iodine.^89^ A section of this publication provides a more quantitative description than Whitehead's review of release pathways and cycling processes. For example, Fig. 7 shows global annual fluxes and steady-state concentrations of iodine throughout various environmental reservoirs.

While the Whitehead^88^ and ATSDR^89^ references provide excellent overviews of iodine in all environmental compartments, a separate reference by Vogt^90^ provides a more in-depth account of iodine in the atmosphere. This reference describes the partitioning of iodine between organic, inorganic, and particulate (iodine solvated in airborne water droplets) forms and tabulates observations of each throughout the world. The text also shows that the photo-dissociation of iodine atoms from organic forms of iodine is highly dependent on speciation (Table 2). The diurnal lifetimes were calculated by scaling the photolysis rate constant by ZA at mid-latitudes throughout a 24 hour cycle during summer and winter. The rate constant is assumed to be zero at night (when ZA = 90). Straight chained *n*-alkyl-iodides are quite long lived whereas branched and multi-halide species have significantly shorter lifetimes. Vogt^90^ also introduces a schematic (without rate constants) mechanism that describes the reactive, photolytic, and heterogeneous exchange of iodine in the atmosphere. This mechanism is a prelude to the separate, extensively cross-referenced paper by Vogt *et al.*^91^

**Table 2:** Properties of select organoiodide compounds including the photolysis rate constants, instantaneous atmospheric lifetimes at a solar zenith angle (ZA) of 40°, diurnal lifetime, and the corresponding ref. 90 and 92–94

Compound	Photolysis rate (s^−1^)	Instantaneous lifetime	Diurnal lifetime (summer–winter)	Ref.
CF_3_I	2 × 10^−5^^a^	14 h^a^	1–3 day^a^	Solomon *et al.*^92^
CH_2_ClI	1 × 10^−4^	3 h	<1 day	Roehl *et al.*^93^
CH_2_I_2_	5 × 10^−3^	3 min	<1 day	Roehl *et al.*^93^
CH_3_(CH_2_)_2_I	7 × 10^−6^	40 h	4–8 day	Roehl *et al.*^93^
CH_3_CHICH_3_	2 × 10^−5^	14 h	1–3 day	Roehl *et al.*^93^
CH_3_I	5 × 10^−6^	55 h	6–12 day	Jenkin^94^

a The data for CF_3_I is calculated for midlatitudes in winter.

### 5.2 Iodine reaction mechanisms

Unlike other halogens (F, Cl, Br), iodine does not play a major role in hydrogen abstraction from volatile organic compounds (VOCs) in the atmosphere. Instead, iodine primarily reacts with ozone (O_3_) to produce iodine monoxide IO˙ radicals. Whereas some subsequent IO reactions lead to the restoration of O_3_ for a “null” cycle, other reaction cycles lead to the catalytic removal of O_3_ from the atmosphere.^90^ The significance of this pathway has led to extensive studies of iodine cycling in the marine boundary layer (MBL) where the biogenic emission of organic iodine from surface algae generates some of the highest concentrations of atmospheric iodine in the world. In 1999, Vogt *et al.* proposed mechanisms to describe these processes.^91^ In 2000, McFiggans *et al.* proposed a variation of the mechanism by adding an IO self-reaction channel in the gas phase and additional aqueous phase pathways to liberate IX (where X represents Cl, Br, or I) back to the gas phase.^95^ Variations of the mechanism (reversible and irreversible uptake into aqueous aerosol) were compared with MBL measurements of IO and a few other iodine species in two locations (Mace Head, Ireland at 53° N and Tenerife Island at 28° N).^95^ Both Vogt *et al.*^91^ and McFiggans *et al.*^95^ are widely cross-referenced in literature about iodine reaction schemes and their effects on O_3_ depletion in the MBL.

### 5.3 Source characterization

Numerous source characterization studies have been conducted to quantify the release of radioiodine from nuclear reprocessing centers in the United States. The purpose of many of these studies was to characterize the impact of emissions to the local environment and more specifically to assess any potential long-term health implications faced by human populations near these facilities. A reference by Kantelo *et al.*^96^ in 1993 is one such study. Because these reports are health related, most have focused on long-term (usually annual) accumulated total emissions and have generally described ^131^I monitoring. These reports can provide some insight into the chemical distribution of iodine emissions. For example, Kantelo *et al.*^96^ reported that 80–90% of iodine released in air effluent from Savannah River Site's H Canyon was characterized as organic with methyl, ethyl, and butyl forms being the most concentrated species observed.

### 5.4 Field measurement studies

Field measurements, *ex situ* of facilities, provide a direct account of the exchange of iodine between different chemical phases and different environmental compartments. These measurements can provide data that are useful for model development and validation. Several studies were identified that targeted characterization of iodine in meteoric water (*i.e.*, rain).^86,97,98^ Moran *et al.*^98^ describe analysis of meteoric water and epiphyte (Spanish moss) samples collected at various locations throughout the United States. The mechanism for iodine content in rainwater is a combination of the heterogeneous nucleation of rain droplets formed around particles that may contain iodine, the chemical adsorption of gaseous iodine material into a droplet, and the physical washout of aqueous and/or nonaqueous aerosols as a droplet falls to the ground. In all cases, the iodine content is a direct result of the atmospheric iodine at the location and specific time of the rain event. Likewise, iodine content in epiphytes is a direct result of atmospheric iodine in the local area. Because these plants do not have a root system, they acquire their nutrients from direct cycling of the air. In contrast to rain measurements, epiphytes provide a long-term integrated collection mechanism that represents average atmospheric conditions for extended periods of time. Although, the absolute iodine concentrations in rain samples were much lower than those reported for epiphyte samples, the ^129^I/^127^I values were consistently in the 1 × 10^−9^ range for both media. These ratios were generally an order of magnitude greater than collections from fresh water and riverine systems in the vicinity suggesting an enhanced atmospheric signal. Moran *et al.*^98^ performed basic transport calculations to attribute these elevations to activity from the Sellafield, England and Cap de La Hague, France reprocessing facilities.

**Fig. 8:**
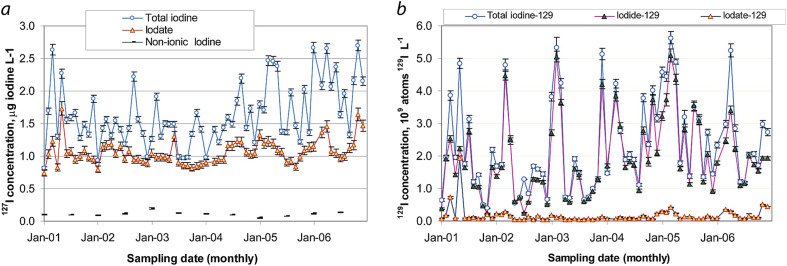
Variations of iodide (I^−^), iodate (IO_3_^−^), non-ionic iodine, and total iodine concentrations in precipitation from Roskilde, Denmark from 2001–2006 for (a) ^127^I and (b) ^129^I. The error bars show a one standard deviation analytical uncertainty. Reprinted with permission from Hou *et al.*^86^ Copyright 2009 American Chemical Society.

Hou *et al.*^86^ reported on another meteoric water study, that showed evidence of a preferential speciation for ^129^I (as iodide) *vs.*^127^I (as iodate) in rain water suggesting a native difference in the primary atmospheric species containing each isotope that was scavenged by the rain (Fig. 8). The authors^86^ suggested that the IO_3_^−^/I^−^ ratio in collected rain could provide an attributable indicator of the origins of air masses that were present during the collection event.

**Fig. 9:**
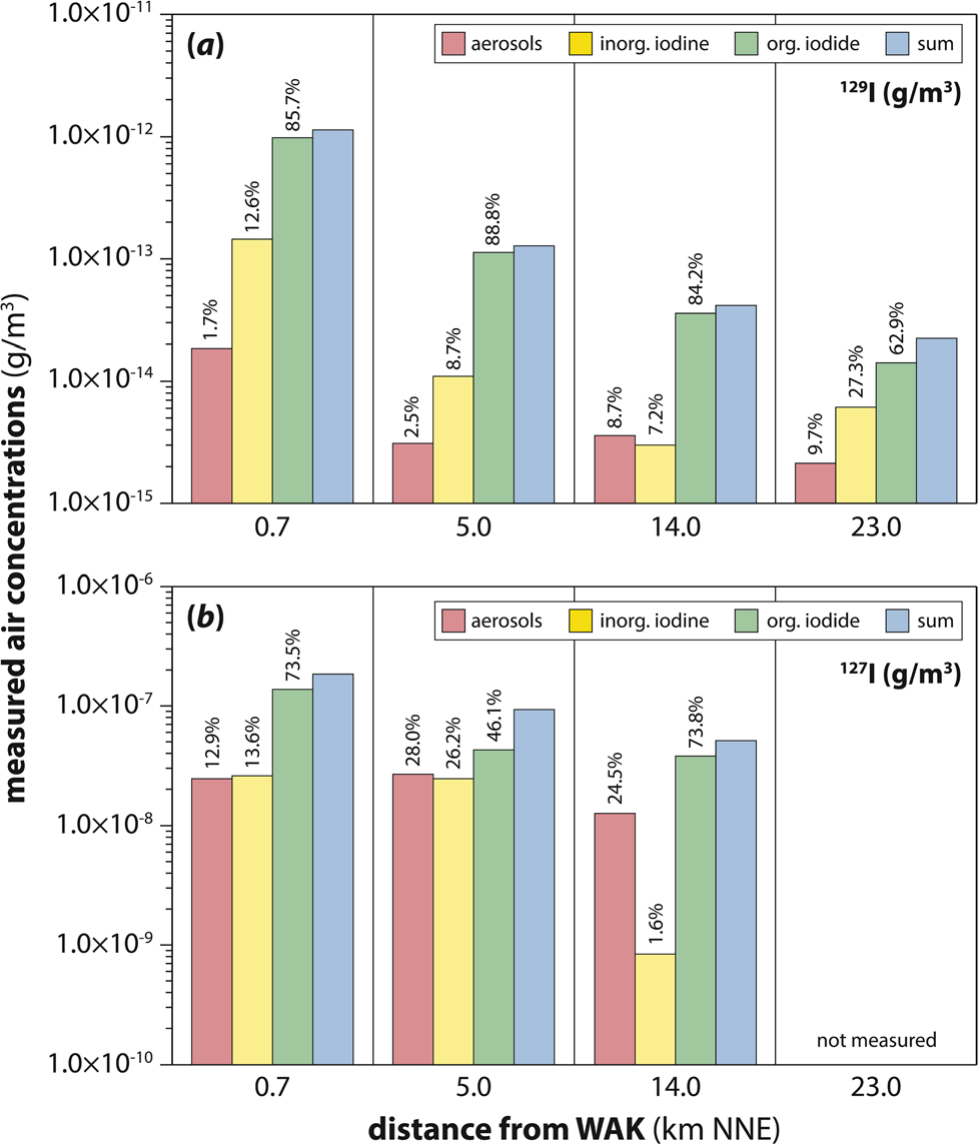
Air concentrations of (a) ^129^I and (b) ^127^I in various chemical forms at four sampling locations NNE from WAK in the dominant downwind direction, as measured between June and September 1987. This figure was modified with permission from Wershofen and Aumann.^105^ Copyright 1989 Elsevier.

The work of Moran *et al.*^98^ and Hou *et al.*^86^ clearly indicate that rain is a major environmental scavenger of atmospheric iodine, regardless of source, and its effects must be included in any atmospheric transport modeling scheme. The 1988–1991 works of Robens, Wershofen, and Aumann^99–106^ reported on environmental iodine at a much closer range to a nuclear reprocessing operation. This reporting is an 8-volume compendium that documents nearly a decade of environmental data from the areas surrounding the Karlsruhe Nuclear Fuel Reprocessing Plant (WAK) in Germany. Chemical distributions (organic, inorganic, and particulate) were characterized at various downwind locations and contrasted with observations in Bonn, Germany, which is upwind of the facility. The distribution of ^129^I clearly favored organic forms whereas the ^127^I was much more distributed across all three chemical forms with only a moderately higher organic content.^105^Fig. 9 shows example data from summer collections between June and September 1987. Similar values were reported for off-gases at the Sellafield facility where 60% of the iodine was organic, 40% was inorganic, and <1% was particulate.^98^

#### 5.4.1 Reactions

As previously discussed, the Vogt *et al.*^91^ and McFiggans *et al.*^95^ environmental fate descriptions for iodine in the MBL are widely cross-referenced sources and virtually no information was found that described characterization over land-based regions remote from the MBL.^91,95^ As a result, these two references were the primary basis for the mechanisms considered in this review. Reaction rates were supplemented with updates reported in the National Institute of Standards and Technology (NIST) and International Union of Pure and Applied Chemistry kinetics databases as well as with data from a few more recent experimental studies.^107–116^

#### 5.4.2 Deposition (wet and dry)

Wet deposition is the loss of gas-phase and/or particulate material (PM) from the atmosphere due to adsorption in rain. The magnitude of the loss is calculated using Henry's law constants for each iodine species. Vogt *et al.*^91^ reported Henry law constants for many iodine species and these values were used directly. Where values were not reported, Henry's law constants were estimated. Model developers reviewed the literature for a wide range of atmospheric species to generate a relationship between Henry law constants and solubility in water. A power function was fit to the data and this relationship was used to fill in data gaps for iodine species.

Dry deposition is the loss of gas-phase material and/or PM from the atmosphere to ground level surfaces (*e.g.*, soil, plants, and buildings). The magnitude of the loss depends on the gravitational settling velocity (for PM only), the flow of the air around or near the surface (calculated from meteorological conditions), the ability of the material to diffuse through air to a surface binding site (calculated from a diffusion coefficient), and the chemical attraction between the surface and the material (calculated from a canopy resistance).^117^

Diffusion coefficients were estimated using the US Environmental Protection Agency Estimation Program Interface Suite 4.10 program.^118^ Dry surface canopy resistance (dry *R*_c_) values were estimated for organic and inorganic species respectively. Organic species are reported to have very slow deposition rates.^88^ As a result, dry *R*_c_ values for these species were set to 5000 to limit the deposition of these materials. For inorganic species, the dry *R*_c_ values were empirically fit to data summarized in a review by Sehmel,^119^ Sehmel^119^ reported deposition velocities of I_2_ ranging from 0.02–26 cm s^−1^ and the majority of these values fell between 0.05 and 7 cm s^−1^.^119^ The reactive atmospheric transport model, which is known as Chemical Calculations using Ordinary Differential Equations (ChemCODE),^120^ was modified to output deposition rates and plume height in order to manually calculate a deposition velocity. A dry *R*_c_ value of 1 injected into ChemCODE produced deposition velocities for a variety of land uses (*e.g.*, forest, grass, water) that were the most consistent with those reported by Sehmel.^119^ This value was then applied to all inorganic iodine species. Wet surface canopy resistance (wet *R*_c_) values were estimated to range from 1 to 5000 and were scaled between species based on their respective solubility values. Table 3 lists the physical property results that were generated by the authors for each iodine species.

**Table 3:** Chemical properties of iodine-containing species

Species	Diffusion coefficient	MW (g mol^−1^)	Canopy resistance (dry deposition)		Henry's law constant (M atm^−1^)	Solubility (g L^−1^)
			Dry *R*_c_	Wet *R*_c_		
DMS^a^	0.1220	62.14	5000	50	0.48	22
CH_3_I	0.1070	141.937	5000	100	0.14	13.8
I_2_	0.0972	253.81	1	5000	3	0.33
HI	0.1270	127.913	1	1	5 × 10^3^	425
HOI	0.1185	143.911	1	10	4.5 × 10^2^	53.5
C_4_H_9_I	0.0789	184.012	5000	5000	6.30 × 10^−2^	0.2
I	0.1315	126.905	1	5000	4.32 × 10^−2^	1.00
IO	0.1220	142.904	1	1	4.50 × 10^2^	450
INO_2_	0.1031	172.91	1	400	0.3	5
OIO	0.0932	158.903	1	150	1	10
IONO_2_	0.0983	188.909	1	1	5000	450
IX	0.1027	207.659	1	700	44.6667	3.51
CH_2_I_2_	0.0873	267.835	5000	5000	2.55	0.833
CH_2_IBr	0.0921	220.834	5000	5000	3	0.759
I_2_O_2_	0.0898	285.808	1	1	5000	450

a DMS (dimethyl sulfide) is a common tracer for testing computational models of atmospheric transport.

#### 5.4.3 Heterogeneous chemistry

Vogt *et al.*^91^ and McFiggans *et al.*^95^ describe particulate iodine in the MBL as water droplets (predominately sea spray) that have dissolved iodine content. Both references use a series of reactions to account for the adsorption, transformation, and release of iodine species from this heterogeneous particulate phase. Because the phase transfer and aqueous reactions have rate constants with different units than those in the gas phase, they cannot be solved simultaneously by a single ordinary differential equation solver like the construct in ChemCODE. As a result, these portions of the reference mechanisms were not used. ChemCODE does have a heterogeneous module that can account for gas phase interactions with particulate matter. The physics-based module allows for a first order treatment of the uptake, reactive loss, and desorption of species onto and from a fixed size distribution of particles with generic composition (*i.e.*, the model does not resolve a differentiation between water, dry aerosols, soot, and other components). The main advantage of using this approach to heterogeneous chemistry was that it did not require a significant modification to the existing ChemCODE architecture during this prototype development to account for what is generally considered to be a minor fraction (<25%) of the total atmospheric iodine content.^88,90,96,98^ This approach is also more flexible if future studies suggest that gaseous iodine has a strong interaction with non-aqueous aerosols that are more typical over land-based transport regimes compared with the MBL. The main disadvantage was that this treatment could not easily leverage the aqueous phase chemistry presented by Vogt *et al.*^91^ and McFiggans *et al.*^95^ Although the particulate fraction of iodine is considered to be small relative to the gas fraction, both sources have shown that this aqueous chemistry can affect speciation within the gas phase.

### 5.5 Meteorology

Iodine is transported globally by atmospheric circulations. For example, increased concentrations of ^131^I released from the damaged Fukushima Daiichi nuclear power plant were detected across the northern hemisphere.^121^ Regardless, both observations and models indicate that the tropics have the highest concentration of iodine as a result of global distribution.^122,123^ Temporally, in the northern hemisphere, iodine concentrations are generally found to be elevated during the summer months.^123–125^

The reactive capability of various inorganic species aids in the formation and growth of particles. In aerosol chamber reactions investigated at the Cosmics Leaving Outdoor Droplets (CLOUD) chamber in the European Organization for Nuclear Research (Conseil européen pour la Recherche nucléaire, CERN), iodic acid (HIO_3_) was shown to have higher particle nucleation and growth rates at low temperature (−10 °C) than sulfuric acid. HIO_3_ particle nucleation rates continued to increase while growth rates were similar to those of sulfuric acid at 10 °C.^126^ Photolysis rates of iodine are likely affected by the cloud field influence on actinic flux.^127^ Additionally, aqueous phase reactions within cloud droplets could play a role in gas to particle conversions.^128^ Models that included HIO_3_ particle formation compared better with particle size distribution measurements than those that only used sulfuric acid.^129^ Additionally, most organic species are short lived and are processed through reactions with OH and photolysis.^130,131^ Droste *et al.*^132^ found that coarse (>1 μm) sea spray and mineral dust particles are more likely to contain IO_3_^−^ because they tend to be more alkaline. However polluted fine mode (<1 μm) particles tend to have more I- and soluble organic iodine due to higher acidity. Increased concentrations of iodine monoxide (IO) were also found in layers of dust plumes off the coast of South America.^133^ The tendency of iodine to adhere to particles can result in long distance transport through the atmosphere^134^ as well as vertically through convection.^135,136^ However, chemical processes within the particles could lead to iodine cycling, which would release the iodine from the particles.^132,137^

Iodine can be deposited to the surface *via* the particle settling (dry deposition) and *via* cloud and precipitation scavenging (washout). Estimated global wet and dry deposition of iodine species is approximately 1.65 Tg per year.^122^ Deposition of iodine has been increasing across the globe since the mid-20^th^ century. These increases have been found in the Greenland ice cap, ice cores from a glacier in France, and in tree rings from spruce trees on the Qinghai-Tibet Plateau.^124,138,139^ Cuevas *et al.* attributed the increase in iodine deposition to increases in anthropogenic ozone pollution which induces increased iodine emissions.^138^ However, Zhao *et al.* attributed increases to human nuclear weapons testing.^139^ Because the deposition of airborne radioiodine onto pasture grass significantly affects the ingestion doses in produce, meat, and dairy, the magnitude of its deposition velocity is extremely important.^140^ Elemental iodine has a significantly higher deposition velocity than other inorganic forms due to its considerable reactivity. Organic forms of iodine are the least reactive and have the lowest relative deposition velocity.

Iodine particles in the atmosphere can act as cloud condensation nuclei that condense cloud water or as ice nuclei that form cloud ice, subsequently leading to precipitation and wet deposition. Falling rain and drizzle drops scavenge additional iodine particles and gases from the atmosphere. On average, precipitation contains 2.0 μg L^−1^ of iodine.^88^ Rainwater iodine concentrations in France varied from 0.8 to 2.7 μg L^−1^. The samples with the highest concentrations were collected in oceanic marine environments and in areas of transition from marine to continental.^125^ However, the lowest lying coastal areas in the United Kingdom were found to have less iodine content in rainwater than was observed 12–35 km inland in an elevated area.^141^ Baker *et al.*^134^ observed wet deposition fluxes to be 2.7 μmol per m^2^ per year at a coastal site near the marine environment.

The removal of iodine through dry deposition occurs when iodine particles settle or when iodine gases adhere to the surface. Estimates of dry deposition of elemental iodine, other inorganic iodine, and iodine particles have been reported to be 1 cm s^−1^, <0.1 cm s^−1^, and 0.1–0.2 cm s^−1^, respectively.^140,142–144^ However, organic forms of iodine, such as CH_3_I, have a significantly lower reported approximate deposition velocities, (0.0001–0.005 cm s^−1^).^140,145^ Dry deposition of elemental iodine depends on the type of vegetation. Tschiersch *et al.*^146^ reported deposition velocities of ^131^I for a variety of leafy vegetables to be 0.16–1.6 mm s^−1^. At a coastal United Kingdom site, Baker *et al.*^134^ reported deposition from aerosol sedimentation as 3.6 to 6.5 μmol per m^2^ per year and direct deposition of CH_3_I as 0.003–0.17 μmol per m^2^ per year.

### 5.6 Geographic considerations

The atmospheric processing and deposition of iodine vary depending on the environment. Iodine in the atmosphere is primarily released from the oceans. A significant portion of the iodine in the oceans resides in ionic forms (I^−^ and IO_3_^−^). Iodine can be oxidized by ozone at the ocean's surface and can be released into the atmosphere as I_2_ and HOI.^147^ Organic forms of iodine (*i.e.*, CH_3_I, CH_2_I_2_, and other halogenated organics) from biological sources are also released from the ocean's surface.^141^ Soluble organic iodine fractions of marine aerosol are largest near the equator and smallest in less biologically productive regions.^148^ Higher humidity near water bodies likely increases reactions between iodine and OH. Marine boundary layer aerosols participate in iodine recycling, in which soluble organic iodine and HOI react and collect onto particles that then release gaseous inorganic iodine.^132,149^ Environments near the coast can be influenced by the transport of iodine from the marine boundary layer.

Because iodine is primarily released from the ocean, terrestrial sources of iodine in the atmosphere are fewer, likely because the low iodine content in crustal rocks limits iodine production from the weathering of rocks.^141^ Iodocarbons can be emitted from terrestrial biogenic processes.^150^ Additionally, iodine is more commonly found in sedimentary rocks and dust.^133,141^ Koenig *et al.*^133^ identified larger concentrations of iodic acid in atmospheric layers containing elevated concentrations of windblown dust off the coast South America. These layers coincided with reduced tropospheric ozone. Iodine could also be released by the burning of vegetation that had previously taken up the iodine.^151^ Thus, local iodine speciation depends on geographic location.

## 6 Summary and conclusions

Before dissolution, the predominate species of iodine in both metal and oxide fuels is expected to be CsI. Depending on burnup, some fraction may form insoluble Pd–Ag iodide compounds in oxide fuels. Less data is available on the forms of iodine in metal fuels. The species of iodine generated during dissolution has been the focus of many research efforts, but owing to the multiple variables, including acid concentration, radiolysis, and sparging, continued research is needed to fully understand this complex system. A large fraction of the iodine partitions to the gas phase during dissolution and, depending on conditions, has the potential to interact with various surfaces in the off-gas system. Some fraction of the iodine is expected to adsorb or absorb to surfaces, although the specific amount remains a multi-variable problem. Abatement is also a well-studied step, but its effectiveness hinges on understanding the quantities and species present in the off-gas system. The iodine that stays in solution after dissolution can move to the extraction step. During extraction, I_2,_ I^−^, and IO_3_^−^ have all been shown to extract at varying degrees to the organic phase. The specific extraction behavior depends on the species present in the aqueous phase, which is variable. Many studies have attempted to elucidate the species and quantities of iodine at various steps in a reprocessing facility; however, the interdependence of the steps necessitates an overall process model to understand the complete system.

## Data availability

No primary research results, software or code have been included and no new data were generated or analyzed as part of this review.

## Conflicts of interest

There are no conflicts of interest to declare.
